# Mediterranean Diet Outcomes Across the Mother–Milk–Infant Triad: A Narrative Review

**DOI:** 10.3390/nu17111760

**Published:** 2025-05-23

**Authors:** Rabia Baglayici, Jadwiga Hamulka, Monika A. Zielinska-Pukos

**Affiliations:** 1 Department of Human Nutrition, Institute of Human Nutrition Sciences, Warsaw University of Life Sciences (SGGW-WULS), 02-776 Warsaw, Poland; rabia.baglayici76@gmail.com (R.B.); jadwiga_hamulka@sggw.edu.pl (J.H.); 2 Department of Nutrition and Dietetics, Institute of Health Sciences, Marmara University, Istanbul 34865, Turkey

**Keywords:** cardiometabolic health, fatty acids profile, infant growth, inflammatory biomarkers, maternal diet, postpartum depression, postpartum weight retention

## Abstract

Nutrition plays a crucial role during pregnancy and lactation, influencing maternal and infant health, as well as human milk (HM) composition. The Mediterranean diet (MED) is recognized worldwide as a well-established healthy dietary pattern. This narrative review aims to integrate and summarize observational and interventional studies investigating the association between the MED and outcomes across the mother–milk–infant triad. A literature search was conducted in the Cochrane, Google Scholar, and PubMed databases, and 22 studies that met the eligibility criteria were included into review. The included studies were categorized into maternal outcomes (*n* = 13), HM composition outcomes (*n* = 9), and infant outcomes (*n* = 3). This narrative review suggests that adherence to the MED may be associated with maternal psychological health and well-being, postpartum weight loss, glucose metabolism, and the fatty acid profile of HM. Moreover, the included studies exhibited notable methodological differences that hindered direct comparisons and limited the generalizability of the findings. Future research should adopt an integrated and standardized methodology for assessing MED adherence, HM collection, and infant outcomes, considering the mother–human milk–infant triad.

## 1. Introduction

Breastfeeding (BF) and human milk (HM) feeding are normative standards in early nutrition, providing tremendous health benefits for infants and mothers across their lifespans [1]. Moreover, recent papers emphasized the adaptive role of HM in various internal (e.g., health status, prematurity, chronobiology) and external factors (e.g., diet and environmental and infectious exposures) that the mother–infant dyad faces [2,3,4]. Hence, the term “mother–milk–infant triad”, as well as the systemic approach to analyzing HM as a complex biological system, was proposed. This approach allows for a better understanding of factors shaping HM composition and how it interconnects with developing infants [2,4].

Maternal diet is a widely studied modifiable factor influencing HM composition. HM production is a priority for the maternal body, and nutritional deficiencies mostly lead to the depletion of maternal stores [2,4,5]. Nevertheless, some of milk’s nutrients were proved to exhibit sensitivity to changes in maternal dietary intake, including fatty acids (FAs) profile, some water-soluble vitamins, and carotenoids [2,5,6]. However, results for other nutrients and bioactives are relatively scarce and inconclusive due to methodological differences and challenges, among others [2,5,7]. Recently, overall dietary patterns, instead of focusing only on single nutrients or foods, were recommended in nutrition research, as they reflect diet complexity and interactions between nutrients [7,8]. Various dietary patterns were linked with adverse (e.g., Western diet) or beneficial (e.g., Mediterranean or Dietary Approaches to Stop Hypertension diet (DASH)) health outcomes due to their unique dietary profile [9].

The Mediterranean diet (MED) is based on low-processed foods such as vegetables, fruits, legumes, herbs, nuts, olive oil, seafood, and fish, as well as whole grains, along with limited consumption of full-fat dairy products, meat (especially red and processed), and alcohol [9]. A substantial body of evidence has linked MED with a lower risk of noncommunicable diseases, such as overweightness and obesity [10], cardiovascular disease [11], metabolic syndrome (MetS) [12], and several types of cancers [13]. In addition, it is also beneficial for cognitive performance, mental health, and well-being across the lifespan [14,15,16,17]. Moreover, MED has been revealed to protect against the development of several adverse pregnancy outcomes, including gestational diabetes, alteration of intrauterine growth, and premature births [18]. Furthermore, it was suggested that maternal MED during pregnancy may support further children’s health [19], yet the current evidence is inconclusive. To date, various studies have investigated the role of MED during the postpartum period, mostly in the context of HM composition [20].

Hence, this review aimed to integrate and summarize observational and interventional studies investigating the association between the MED and outcomes across the mother–milk–infant triad while identifying potential gaps and directions for further studies. We chose a narrative approach over a systematic review due to the expected substantial heterogeneity in study designs, populations, and outcomes. In addition, our goal was not to draw definitive conclusions but to provide an overview of the current state of knowledge, highlight the effects of the MED on the mother–milk–infant triad, and indicate areas where further studies are needed.

## 2. Materials and Methods

This narrative review was conducted using PubMed, Google Scholar, and the Cochrane Library databases from 10 January 2025 to 31 January 2025, including interventional and observational studies evaluating the Mediterranean diet’s outcomes among mother–human milk–infant triads published in English over the past 20 years. Detailed inclusion and exclusion criteria are shown in Table 1. To identify relevant studies, the following combinations of keywords were used: (“mediterranean diet” OR “mediterranean diet adherence” OR “mediterranean dietary pattern” OR “mediterranean index” OR “mediterranean score” OR “med diet”) AND (“lactation” OR “lactating” OR “breastfeeding” OR “postpartum”) AND (“breast milk” OR “breastfeeding” OR “human milk” OR “colostrum” OR “mature milk” OR “donor milk” OR “transitional milk”) AND (“maternal” OR “maternal outcomes” OR “health”) AND (“infant” OR “neonatal”).

**Figure 1:**
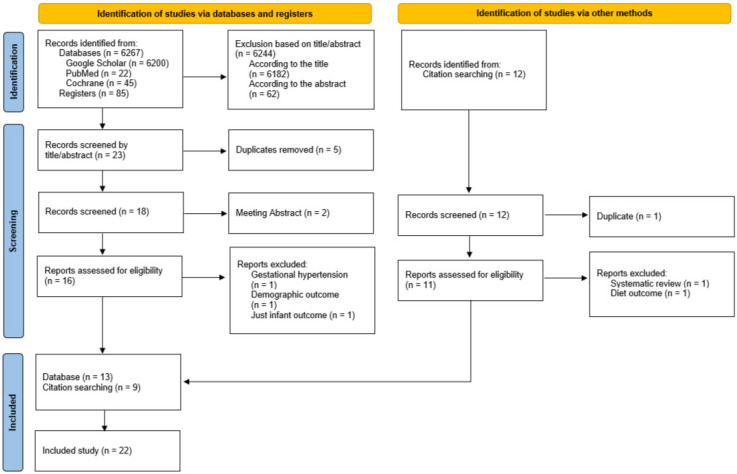
A flow diagram of the records is included in the narrative review.

The search and eligibility process is presented in Figure 1. The titles and abstracts of the studies were screened by a researcher (R.B.) to determine their eligibility for inclusion. A second researcher (M.A.Z.-P.) independently verified the selection.

Due to high heterogeneity in eligible studies, we employed a narrative approach to synthesize key findings. The outcomes of interest were maternal (psychological, well-being, anthropometry, and metabolic outcomes), lactation and HM (breastfeeding and HM composition), and infant-related (growth and metabolic outcomes) parameters; we relied on information in tables, figures, and the interpretation of the results of each included paper to perform a cross-study synthesis. Then, relevant data were extracted, including (1) study characteristics: authors, year of publication, study design, sample size, country of origin, and sample characteristics; (2) exposure evaluation and measures: dietary assessment or intervention details, MED diet assessment (index and its components), and timing of dietary assessment; and (3) outcome measures: measures, methods, timing of assessment, and covariates included in statistical analyses. Extracted information was organized thematically into maternal, HM, and infant outcomes. We did not evaluate the quality, as this review aimed to integrate and summarize observational and interventional studies and identify potential directions for future studies, not to synthesize systematically and draw clear conclusions on the current state of knowledge.

## 3. Results

**Figure 2:**
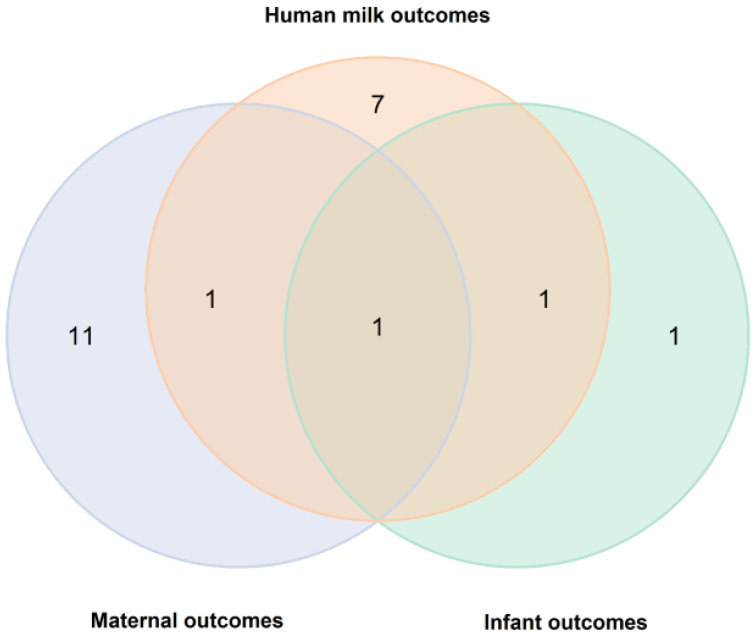
A Venn diagram of analyzed outcomes in publications included in the review.

As a result, 22 studies were included in the narrative review. The included studies were categorized based on maternal (*n* = 13 studies), HM (*n* = 9 studies), and infant outcomes (*n* = 3 studies). Among them, only one study analyzed outcomes in all elements of the mother–milk–infant triad [21], one in the mother–milk dyad [22], and one in the milk–infant dyad [23] (Figure 2).

### 3.1. Mediterranean Diet and Maternal Outcomes

#### 3.1.1. Psychological Health and Well-Being

Studies evaluating the associations between the MED and maternal psychological and well-being outcomes (*n* = 3, [24,25,26]) are presented in Table 2. Two studies found a negative association between high adherence to the MED and postpartum depression [25,26]. A study conducted in Spain reported no significant effect of the MED on stress levels or postpartum depression but found an improvement in overall positive attitude, which may be limited due to a lack of adjustment of statistical analysis (Table S1) [24]. There were differences in the timing of dietary assessment among the studies. Gila-Diaz et al. [24] assessed adherence at 28 days postpartum, Flor-Alemany et al. [25] at 16 weeks of gestation, and Papadopoulou et al. [26] between 3 and 6 months postpartum. Additionally, all studies adapted various indices for MED assessment [27,28,29], varying in dietary components used for its calculation (Table S1). These variations in exposure assessment may have contributed to the inconsistent findings regarding the association between the MED and postpartum depression by affecting the degree to which participants adhered to the diet over time [24,25,26].

#### 3.1.2. Anthropometry and Body Composition

Table 3 summarizes studies evaluating the association between adherence to the MED and maternal anthropometric outcomes (*n* = 8 [21,30,31,32,33,34,35,36]).

Three studies reported a negative association between adherence to the MED and maternal body weight [21,30,34]. Additionally, one study found a negative relationship between adherence to the MED and body weight change [36]. However, two studies did not identify any significant association [31,33]. Additionally, one study found an inverse relationship between postpartum weight retention (PPWR) and two different types of MED indices (alternate MED (aMED) and Lebanese Mediterranean diet (LMD)) at 2 and 6 months after childbirth [32].

The potential association between MED adherence or intervention and maternal body mass index (BMI) was assessed in six studies [21,30,31,33,34,36]. Three studies concluded that adherence to the MED was associated with a reduction in BMI levels [21,30,34]. In contrast, three studies did not observe any significant association [31,33,36].

Two studies reported a reduction in body fat percentage among mothers with high adherence to the MED [30,33]. Furthermore, another study found a negative association between adherence to the MED and fat mass (FM) [35]. In contrast, two studies did not identify any significant relationship [34,36]. Regarding the fat mass index (FMI), two studies reported a negative association [21,33]. Lean body mass was positively associated with adherence to the MED in one study [35], whereas another study found no significant relationship with the lean body mass index [21]. In the only study investigating FM distribution, mothers with high adherence to the MED exhibited a reduction in the android FM and the android/gynoid FM ratio [35].

There were a total of four studies on waist circumference (WC) [30,31,34,36]. Two studies reported a reduction in WC among mothers adhering to the MED [30,34], whereas the other two found no significant association [31,36]. In two studies, a significant reduction was observed in mothers with a WC exceeding 89.5 cm, a risk factor of MetS [34,36]. Hip circumference was found to decrease in only one study [30]. Additionally, an investigation into the waist-to-hip ratio revealed a reduction in this parameter among mothers adhering to the MED [30].

Differences in anthropometric outcomes may be attributed to variations in assessment tools and timing in body composition evaluation, as well as differences in study design, whether cross-sectional or interventional. For example, included studies varied in indices used for MED assessment, including food groups used for its calculation or geographical adaptations [28,37,38,39,40,41] (Table S1). Three of the included studies were based on the same MED index (MEDAS [42]) [31,34,36] but differed in study group characteristics and timing of outcome assessment, which may have contributed to discrepancies in findings.

#### 3.1.3. Metabolic Outcome

Table 4 presents studies evaluating associations between the MED and maternal metabolic outcomes. A total of seven studies examined the effects of maternal adherence to the MED or MED interventions on biomarkers in maternal blood serum or plasma, including FAs, lipid profile, glucose, insulin, inflammatory biomarkers, leptin, blood pressure, and components of MetS [21,30,31,34,36,43,44].

Two studies reported that maternal adherence to the MED was associated with reduced cholesterol levels [21,34]. Moreover, the anthropometric outcomes of these studies supported these findings. In the studies conducted by Melero et al. and Sims et al., reductions in weight, BMI, and WC levels were observed [21,34], whereas no significant changes were reported in the studies by de la Torre et al. and Martín-O’Connor et al. [31,36].

Regarding lipid profile, two studies identified a negative association between high-density lipoprotein cholesterol (HDL-C) levels and adherence to the MED [21,34], while two others found no relationship [31,36]. Furthermore, three studies reported that high adherence to the MED was linked to lower low-density lipoprotein cholesterol (LDL-C) levels [21,34,36], whereas one study found no association [31]. In parallel with the reduction in LDL-C levels, the anthropometric findings of these studies also indicated decreases in weight and WC [21,34]. However, in the study where no association with LDL-C was found, no significant changes in weight or WC were observed [31]. Additionally, when examining the relationship between maternal adherence to the MED and triglyceride (TG), two studies found no association. These findings are consistent with the lack of changes in the anthropometric outcomes of these studies [31,36]. Melero et al. reported a reduction in apolipoprotein B levels at 3 months and 3 years postpartum [34].

One study identified a positive association between maternal adherence to the MED and leptin levels [44], while another study found no significant relationship [21].

In the study conducted by Stendell-Hollis et al., high adherence to the MED was associated with increased levels of linoleic acid (LA), alpha-linolenic acid (ALA), ω-3, and the ω-3/ω-6 ratio, while the ω-6/ω-3 ratio decreased [30]. These findings are consistent with the reductions in body fat percentage observed in the study by Stendell-Hollis et al. [30].

Che et al. demonstrated that high maternal adherence to the MED was positively associated with triacylglycerols (C54:8, C54:9, C56:9, C58:9, C58:11, C56:10, C60:12, C58:8, C56:8, C56:7, C58:10, C58:7, and C52:7), phosphatidylcholines (C40:10, C40:9, and C40:6), PC plasmalogen (C36:5 PC plasmalogen-A), phosphatidylserine (C34:0), phosphatidylethanolamine (C40:6), methionine sulfoxide (an amino acid), and vitamin A levels, while it was negatively associated with threonine (an amino acid), tropane, and adenosine (nucleotides) levels [43].

There were four studies examining glucose and insulin biomarkers [21,31,34,36]. De la Torre et al. found no association between maternal adherence to the MED and glucose, insulin, homeostatic model assessment for insulin resistance (HOMA-IR), or hemoglobin A1c (HbA1c; glycated hemoglobin) levels [31]. Similarly, Sims et al. reported no significant relationship with insulin levels [21]. In the study by Melero et al. [34], no changes in glucose levels were observed three years postpartum; however, at three months postpartum, a negative association was found with glucose, insulin, and HOMA-IR levels. Additionally, a negative association with insulin and HOMA-IR levels was also observed three years postpartum. In this study, improvements in insulin resistance were consistent with reductions in weight, BMI, and waist circumference [34]. Furthermore, a study conducted in Spain reported a reduction in impaired fasting glucose (FG) levels [36].

Three studies examined inflammatory biomarkers [21,30,34]. In the study by Melero et al., C-reactive protein (CRP) levels were found to decrease both at 3 months and 3 years postpartum. This finding was consistent with the reductions in weight, BMI, and WC observed in the same study [34]. Another study reported no association with interleukin-6 (IL) levels [30]. Similarly, Sims et al. found no significant relationship between maternal adherence to the MED and CRP, IL-6, IL-8, or tumor necrosis factor α (TNF-α) levels [21].

Three studies examined blood pressure levels [31,34,36]. Two of these studies found no association between maternal adherence to the MED and systolic blood pressure. These findings are consistent with the lack of significant BMI and WC changes observed in the same studies [31,36]. Regarding diastolic blood pressure, Melero et al. reported a negative association [34], whereas another study found no significant relationship [36]. A similar pattern was observed for MetS [34,36]. The observed reductions in weight, BMI, and WC, alongside the decrease in blood pressure in the study by Melero et al., support the beneficial effects of the MED on cardiometabolic health [34].

Studies varied in the indices used for MED assessment, including its food groups used for their calculation [37,39,42,45,46,47] (Table S1). Despite the three studies that were based on MEDAS [31,34,36], they introduced index adaptations and varied in assessment timing, which introduced differences in results.

### 3.2. Mediterranean Diet and Human Milk Outcomes

The effects of maternal adherence to the MED on HM outcomes are presented in Table 5. The majority of studies (with one exception [48]) analyzed HM samples from the first six months of lactation. In the study conducted by Antasouras et al., mothers with high adherence to the MED in the last trimester of pregnancy had a higher rate of exclusively breastfeeding their infants for at least four months [49].

A total of two studies investigated the macronutrient composition of HM [21,23]. A study conducted in 2023 found that mothers with high adherence to the MED had increased protein content and decreased TG levels in their milk [23]. In contrast, the study by Sims et al. found no association between adherence to the MED and HM energy value or macronutrient composition [21].

Three studies investigated the lipid profile of HM [20,50,51]. Krešić et al. reported that mothers with high adherence to the MED had increased HM levels of docosahexaenoic acid (DHA), LA, palmitic acid, and oleic acid (OA). At the same time, no changes were observed in arachidonic acid (AA) levels [50]. Codini et al. found a positive association between adherence to the MED and the levels of saturated fatty acids (SFA) and polyunsaturated fatty acids (PUFA) in HM [51]. In another study conducted in Italy, a decrease was observed in SFA, palmitic acid, stearic acid, AA, the ω-6/ω-3 ratio, and the LA/ALA ratio. Conversely, an increase was noted in monounsaturated fatty acids (MUFA), OA, erucic acid, ω-3 PUFAs, ALA, eicosapentaenoic acid (EPA), DHA, docosapentaenoic acid, and the DHA/AA ratio [20].

Two studies investigated the micronutrient composition of HM [44,48]. Sánchez et al. examined selenium levels in HM and found that they were higher in mothers adhering to the MED. However, no significant changes were observed in sodium, potassium, calcium, the calcium-to-phosphorus ratio, magnesium, or iron levels [48]. Additionally, a study conducted in Poland reported a positive association between adherence to the MED and calcium and zinc levels in HM, while no relationship was found for iron and phosphorus levels [44]. These studies both evaluated HM minerals using the ICP-MS method but differed in the timing of HM collection and assessment of adherence to the MED [44,48]. In the study by Zielinska-Pukos et al., HM was collected in the first month of lactation [44], whereas Sánchez et al. carried out the study over a broader postpartum period (0.8–59 months postpartum) [48], which may contribute to variation in HM composition [44,48].

Five studies investigated the relationship between maternal adherence to the MED and the bioactive compound content of HM [21,22,23,51,52]. Three studies reported a positive association between adherence to the MED and antioxidant levels. The timing of the MED assessment was unclear in all three studies conducted [23,51,52]. In their study, Codini et al. found that the antioxidant potential in the HM of mothers who followed the MED was not affected by preterm or term birth [51]. Additionally, total phenolic compound levels were found to be increased. Moreover, no differences in total phenolic acid or antioxidant activity were observed across the three stages of HM (colostrum, transitional milk, and mature milk). In contrast, significant differences in antioxidant properties were found between HM and infant formulas [52]. Two studies conducted by Sims et al. and Zielinska-Pukos et al. focused on obese, lactating mothers and reported a negative association between HM leptin levels and adherence to the MED [21,22]. Furthermore, the study by Sims et al. reported a negative association between the MED and IL-8 and TNF-α levels in HM, while no significant relationship was observed for human milk oligosaccharides, insulin, IL-6, or CRP levels [21].

Similarly to previous outcomes, studies varied in analyzing HM and lactation outcomes and varied in methods of MED indices analysis [37,39,42,46,47,53] (Table S1). Studies varied in food group building indices, while two studies did not calculate a specific MED score; as stated, the whole group exhibited a moderate-to-high adherence to MED [50,51] (Table S1).

### 3.3. Mediterranean Diet and Infant Outcomes

The effects of maternal adherence to the MED on infant outcomes are presented in Table 6. In total, three studies examined this relationship [21,23]. The study by Karbasi et al. investigated urinary antioxidant levels in infants and found a positive association [23]. Another study examined infant anthropometry and body composition but found no significant relationship between maternal adherence to the MED and weight-for-age, length-for-age, weight-for-length z-scores, fat-free mass index (FFMI), or fat mass index (FMI levels [21]. Similarly, Grabowski et al. found no association with weight-for-age and length-for-age parameters; however, flank skinfold thickness was negatively associated [54]. The indices used to assess adherence to the MED varied across studies, including differences in the food groups considered in their calculation (Table S1). In all three studies, MED scores were calculated based on different dietary indices [37,39,46]. Additionally, there were notable differences in study design, sample size, and the timing of outcome assessments.

## 4. Discussion

In this narrative review, we summarized studies examining the potential effects of the MED during pregnancy or lactation on maternal, infant, and HM outcomes. Existing studies most often examined maternal outcomes during the postpartum period. They suggested that the MED was linked to maternal psychological health and well-being, LDL, and HOMA-IR and potentially to maternal anthropometry, TGs, cholesterol, HDL-C, and blood pressure. HM and lactation outcomes were the second most often investigated area. Up-to-date studies linked MED to the HM lipid profile, antioxidant, and phenolic components, whereas associations with inflammatory biomarkers, macronutrients, and micronutrients in HM remain less clear. To date, a limited number of studies have investigated infant outcomes, mostly anthropometric development, which remain inconclusive. It should be emphasized that studies were characterized by high heterogeneity and mostly focused on one angle of the mother–milk–infant triad, while MED outcomes across the whole triad were analyzed only once.

### 4.1. Maternal Mental Health and Well-Being

Studies identified in this narrative review suggested that MED may be associated with maternal postpartum mental health [24,25,26], which is in line with studies conducted among non-lactating individuals. It was shown that nutrition plays a crucial role in cognitive processes and mental health. Most studies focused on the relationship between depression and specific nutrients or foods [55,56]. Depressive symptoms were associated with low levels of B vitamins, including folate, riboflavin, pyridoxine, and cobalamin, potentially through mechanisms involving homocysteine levels or monoamine synthesis in the brain [57]. Moreover, vitamin D plays a significant role in brain function and development in women experiencing postpartum depression. However, the extent to which vitamin D is involved in increasing estradiol levels, reducing NF-kB activation in macrophages, and/or decreasing the production of pro-inflammatory cytokines remains unclear. Overall, there is evidence suggesting an association between 25[OH]D levels and postpartum depression [58]. Similarly, ω-3 PUFAs, with ALA, EPA, and DHA as key components, play significant roles in brain function and activity and have been suggested to contribute to the development of depression [59]. The MED provides these essential nutrients and has been shown to reduce depressive symptoms and improve remission rates in the general population [60], as well as enhance well-being in women of reproductive age [61]. Likewise, it may have a protective effect against postpartum depression [62]. In a study conducted by Chatzi et al., adherence to a diet rich in vegetables, fruits, legumes, nuts, dairy products, fish, and olive oil during pregnancy was found to have a protective effect against postpartum depression [63]. Additionally, the consumption of fish and/or ω-3 PUFAs was associated with a reduced risk of postpartum depression [64,65]. Dietary patterns during pregnancy that include nuts, fruits, and seafood were also associated with a lower prevalence of postpartum depression [66]. Furthermore, the antioxidant and anti-inflammatory properties of fruits, vegetables, and nuts, along with their content of B vitamins essential for the synthesis of tryptophan and 5-hydroxytryptamine, were found to reduce the risk of postpartum depression [65,66]. Moreover, the effectiveness of the MED in depression was attributed to its high polyphenol content, with various polyphenols demonstrating a reduction in depressive symptoms and providing a beneficial therapeutic effect for depression [67].

### 4.2. Maternal Anthropometric Outcomes

Pregnancy is associated with physiological weight gain and adipose tissue accumulation to satisfy elevated energy requirements during lactation [68]. It has been reported that 75–80% of pregnancy weight gain is lost within 2–6 weeks postpartum, but it could be affected by various factors, including pre-pregnancy BMI and BF practice [30,69]. Excessive gestational weight gain (GWG) and PPWR are potential risk factors for obesity-related disorders, including insulin resistance, MetS, and cardiovascular diseases [32,70,71]. Hence, it is essential to identify potential modifiable factors protecting against excessive GWG and PPWR. Dietary intervention could play a crucial role in weight and body composition management [72]. In addition, pro-healthy dietary patterns, such as the MED, emerge as a potential strategy to maintain a healthy weight and mitigate obesity-related health risks. In particular, the traditional Mediterranean dietary pattern, which is rich in vegetables, olive oil, and nuts, is characterized by low energy density, low glycemic load, high water and fiber contents, and an emphasis on plant-based foods. These attributes may enhance satiety, prevent excessive food intake, and contribute to healthy weight management during pregnancy, preventing excessive weight gain [73,74,75]. Moreover, higher adherence to the MED before pregnancy has been associated with lower BMI increases during gestation, while adherence during pregnancy has been shown to support appropriate weight gain [75]. Interestingly, a study conducted on South African women found that adherence to a diet rich in whole grains, legumes, and vegetables was associated with a lower risk of excessive GWG, particularly in women with a normal weight [76]. A meta-analysis including four randomized controlled trials and 2277 participants confirmed that the MED is associated with a significant reduction in gestational weight gain (standard mean difference −0.15, 95% CI –0.26 to –0.05, *p* = 0.004) [77]. Moreover, a study combining diet and physical activity has been associated with significantly lower GWG [78]. According to studies conducted worldwide, the mean PPWR measured at 6 months was reported as 2.1 kg in Taiwan, 3.12 kg in Malaysia, and 3.3 kg in the United States [79,80,81]. Lower dietary quality during pregnancy has been shown to be associated with a higher likelihood of a significant PPWR of 5 kg or more [82]. Further research is needed to clarify the role of dietary interventions in PPWR.

MED can also affect different measures of adiposity, such as WC and body composition. In the general population, a higher MED score has been associated with lower WC gain [83,84]. In the SUN cohort, participants with a higher baseline MED score exhibited a lower WC after a 6-year follow-up period [85]. PPARγ is a gene involved in adipocyte differentiation, lipid storage, and regulation of body composition. Furthermore, the MED has been suggested to reduce WC by counteracting the adverse effects observed in high-cardiovascular-risk carriers of the PPARγ 12Ala allele related to fat accumulation [86]. It has been suggested that the high levels of PUFAs and MUFAs in the MED may activate the PPARγ protein and potentially modify the effect of the Pro12Ala substitution on receptor activity [87].

### 4.3. Maternal Metabolic Health

During pregnancy, maternal metabolism undergoes changes that promote energy storage and nutrient transfer into the fetus. This is related to increased visceral fat, insulin resistance, and lipid levels. BF helps restore metabolic balance and reduce the risk of further metabolic diseases [88]. Maintaining an adequate and balanced diet may contribute to resetting maternal metabolism during lactation. In this context, the MED may play a significant role in metabolic regulation, including glucose metabolism [18]. The beneficial effects of the MED on glucose metabolism may be attributed to the high intake of dietary polyphenols present in its key components, such as extra-virgin olive oil and nuts. These effects may improve metabolic processes by reducing insulin resistance, stimulating insulin secretion, activating insulin receptors, regulating glucose release, and enhancing glucose uptake in insulin-sensitive tissues [89]. The MED has been shown to reduce high blood pressure in at-risk populations, and women consuming diets with low MED adherence were found to have lower FG levels at delivery [90].

Furthermore, the MED’s rich content of unsaturated fats, fiber, and antioxidants has been suggested to play a significant role in modulating inflammation [30]. Greater adherence to the MED has been significantly associated with lower circulating concentrations of inflammatory markers [91]. A significant negative correlation has also been observed between high adherence to the MED during pregnancy and CRP levels [92]. However, Stendell-Hollis et al. found no association between the MED and inflammatory markers, other than TNF-α, in lactating women [30]. These findings were further supported by the study conducted by Sims et al. [21].

### 4.4. Human Milk Outcomes

A mother’s diet influences HM composition through various metabolic pathways [93]. Specifically, the maternal intake of FAs and fat- and water-soluble vitamins (including vitamins A, C, B6, and B12) can directly reflect their concentrations in HM [20,93,94]. The MED is characterized by an anti-inflammatory FAs profile caused by a low intake of saturated ω-6 and trans FAs and a moderate intake of plant-based and marine-derived ω-3 PUFAs [50]. It has been shown to significantly affect the FA profile and antioxidant factors in HM [95]. A study conducted in Italy found a significant correlation between maternal dietary SFA and MUFA levels with those in transitional milk, while PUFA showed the highest correlation in mature milk [96]. Similarly, a study conducted on Mediterranean women showed that both total fat intake and PUFA intake were strongly associated with DHA levels in HM during the first month of lactation [97]. These results are believed to stem from the consumption of olive oil, which is rich in MUFAs, especially OA, as a primary fat source in the MED [20].

Maternal overweightness and obesity can negatively affect HM composition [22,98]. The most evident alterations are the pro-inflammatory FAs profile and elevated levels of adipokines, such as leptin [98,99]. Two recent studies have shown that the MED can mitigate elevated milk leptin in mothers with an excessive body mass [21,22]. In an intervention study by Sims et al., this could be related to observed weight loss and decreased FMI. In addition, they observed a decrease in HM pro-inflammatory chemokines [21].

HM plays a crucial role in the protection against oxidative stress diseases during infancy due to its infancy-unique antioxidant properties [100]. Some of the HM antioxidants, such as antioxidant vitamins (e.g., C and E), selenium, polyphenols, and carotenoids, are derived from the maternal diet [101,102]. The MED has been shown to be efficient in improving the total antioxidant capacity of healthy, non-lactating adults [23,103] due to the high abundance of vegetables, fruits, nuts, and whole grains. Studies included in this review have shown that during lactation, adherence to the MED results in an increase in HM selenium [48] and the total antioxidant capacity of HM [23].

### 4.5. Infant Outcomes

During exclusive BF, infants rely solely on HM’s nutrients and bioactivity [93,103]. Moreover, even when solids are consumed, HM satisfies a significant part of infant and toddler nutritional requirements [104]. Hence, all factors influencing HM composition can indirectly affect infant growth and development [93,105]. Existing studies have primarily focused on the MED effect on birth and early childhood outcomes [18,106,107]. Unfortunately, few studies have examined maternal MED on outcomes in breastfed infants [21,23,54]. Two studies analyzed and failed to confirm its effect on infant anthropometry [21,54]. On the other hand, various studies have shown them to have a positive influence on intrauterine growth and fetal development [18,105,108]. High adherence to the MED has been associated with a reduced risk of preterm birth and small-for-gestational-age infants, as well as with lower rates of childhood obesity, leptin levels, and blood pressure [106,107]. Furthermore, it was shown that the MED during pregnancy benefits further cognitive and behavioral outcomes [105,109,110], but no study investigated its effect in the mother–human milk–infant triad. Nevertheless, it is highly plausible that the maternal MED will positively shape infant neurocognitive development, as it increases HM PUFAs [20,50,51]. PUFAs, especially DHA and AA, are essential for fetal and neonatal growth and neurodevelopment [50,111], and it was shown that their milk levels are related to infant motor development [112]. One of the studies included in this review has shown that a maternal MED boosts infant urine antioxidant potential through the improvement of HM antioxidant capacity [23]. Nevertheless, the long-term effects of these changes in HM composition on infant health and development remain largely unknown, underscoring the need for further longitudinal research.

### 4.6. Methodological Aspects and Recommendations for Further Studies

Studies included in this review were characterized by high heterogeneity, which can explain the ambiguity of the results. Firstly, studies had various designs, including cross-sectional [20,22,23,24,26,33,44,48,49,50,51,52], prospective [32,43,54], and intervention [21,25,30,31,34,35,36] studies that affected the quality of the results. Secondly, studies varied in the exposure assessment. Some studies analyzed MED adherence based on food frequency questionnaires and various a priori indices (sometimes modified or geographically specific), while others utilized a MEDAS questionnaire [31,34,36,48], or imported methodological details were omitted (Table S1). All of the dietary indices used for the assessment of MED adherence included vegetables and legumes. One study did not specify the source of animal proteins, nor did it evaluate fish and/or seafood intake [32]. In addition, studies varied in exposure period, as some of the studies analyzed the effect of the MED in pregnancy [25,31,32,34,35,36,49] and others in the postpartum period [20,21,22,23,24,26,30,43,44,50,54]. Similarly, the type of intervention varied from educational dietary intervention [31,34,36], through the additional portion of extra-virgin olive oil and nuts [30,31,34,36], to two ready-to-eat meals provided by research staff [21]. In addition, the included studies encompassed diverse populations varying in geographical, cultural, socioeconomic, and health contexts (e.g., term vs. preterm, normal weight vs. overweight/obese), which may influence dietary and BF practices, as well as health outcomes. These differences make it harder to compare, synthesize, and generalize results, and it can lead to different effect estimates [8]. Thirdly, the assessment of outcomes significantly varied, including the timing of assessment (from a few hours to 3 years postpartum) to research methodology (e.g., in maternal anthropometric analysis or HM collection). It should be highlighted that studies analyzing HM composition should adopt a rigorous sample collection protocol to minimize variations in its composition that may bias study results [113].

Ideally, further studies should include dietary intervention across the pregnancy and postpartum periods to evaluate windows of sensitivity and the effect of maternal MED on offspring outcomes. If intervention design is impossible, long-term observational studies with various time points of exposure assessment are recommended. Finally, studies focusing on outcomes in the postpartum period among lactating women should also include HM and breastfed infants with the investigation of long-term effects. Taking into account the multifractional determinants of offspring development, studies should carefully determine potential covariates and consider the mother–infant–human milk triad. Moreover, considering the complexity of lactation, HM composition, and dietary and growth determinants, further studies should carefully target study populations in terms of maternal and infant factors, including ethnicity, socio-economic status, health status, and BF practices. Additionally, it is imperative to incorporate underrepresented groups, as the effects observed can significantly differ based on health (e.g., maternal overweight/obesity, preterm/term delivery), ethnicity, and other critical variables (for example, socioeconomic status and education level) that may affect exposures and outcomes. Understanding the effect of the diet across the mother–milk–infant triad is crucial, as diet-induced changes in HM composition can indirectly influence infant outcomes. Taken together, employing this approach should allow for the obtaining of stronger clinical evidence on the role of MED across the mother–milk–infant triad and help to elucidate the mechanisms underlying these effects.

## 5. Conclusions

This narrative review summarizes studies investigating MED outcomes across the mother–infant–human milk triad. Adherence to the MED during pregnancy and lactation has been shown to be associated with improved maternal psychological health and well-being, weight loss, glucose metabolism, and HM FA profile. Some studies have suggested that MED may be associated with maternal metabolic health and HM components other than FAs, as well as offspring development, but these findings remain scarce and inconsistent and rarely address the mother–milk–infant triad holistically. There is a significant gap in literature examining long-term infant outcomes, such as growth, neurodevelopment, and health status, in relation to maternal MED adherence during lactation. Another major limitation of existing literature is the considerable methodological heterogeneity, including differences in study design, exposure and outcome assessments, and population characteristics. This limits the comparability and generalizability of findings and precludes establishing solid conclusions regarding the role of the MED in shaping outcomes across the maternal–HM–infant triad.

Therefore, there is a clear need for future research employing standardized, integrated approaches that consider the mother–milk–infant triad as a whole, utilize validated dietary assessment tools, and adopt rigorous protocols for HM collection and infant outcome evaluation. Given the importance of infant health and development, future studies should prioritize the rigorous and comprehensive evaluation of infant outcomes alongside maternal and milk parameters. A holistic, triad-based research strategy will be critical for generating robust clinical evidence on the role of the MED in maternal and child health and for elucidating the mechanisms through which maternal nutrition influences HM composition and, ultimately, infant outcomes.

## Figures and Tables

**Table 1:** Inclusion and exclusion criteria for screening studies.

Type of Criteria	Criteria
Inclusion	observational or intervention studies investigating adherence to or MED intervention during pregnancy or lactation evaluation of maternal, breastfeeding, and/or infant outcomes among breastfeeding mother–infant dyads articles in English articles published between January 2005 and January 2025
Exclusion	outcomes measured in non-breastfeeding mothers and/or formula-fed infants outcomes measured exclusively during pregnancy or delivery no data on breastfeeding status or human milk composition animal studies reviews, systematic reviews, or meta-analyses study protocols conference abstracts articles with no full text available

MED—Mediterranean diet.

**Table 2:** Studies evaluating associations between the Mediterranean diet and maternal psychological and well-being outcomes.

No.	Author and Year	Study Design, Name	Study Size, Country	Sample Characteristic (Special Features, Age)	Dietary Assessment/ Intervention, Timing	Mediterranean Diet Assessment	Outcome (Measures, Methods, Timing of Assessment)	Effect of the Mediterranean Diet
1	Gila-Díaz et al., 2021 [24]	Cross-sectional study	*n* = 55 Spain	*n* = 30 term deliveries *n* = 25 preterm deliveries 34.0 (31.5–37.0) y	AP-Q (graphic representation of MED diet questionnaire) 28 d postpartum	AP-Q (11 components)	Stress (PSS); overall positive attitude (LOT); postpartum depression (EPDS) 2×: 14 d and 6 m postpartum	↑ positivity (14 d and 6 m) ↔ postpartum depression, stress
2	Flor-Alemany et al., 2022 [25]	RCT GESTAFIT	Spain *n* = 85	*n* = 46 exercise intervention *n* = 39 control 33.4 ± 4.2 y	FFQ 16 Hbd	Mediterranean Food Pattern (8 components)	Postpartum depression (EPDS) 6 wk postpartum	↓ postpartum depression
3	Papadopoulou et al., 2023 [26]	Cross-sectional study	Greece *n* = 3941	33.2 ± 5.6 y	3–6 m postpartum	MedDietScore (11 components)	Postpartum depression (EPDS) 3–6 m postpartum	↓ postpartum depression

↔ no association/effect; ↑ positive association/effect; ↓ negative association/effect; AP-Q, adherence to the healthy food pyramid; d, days; EPDS, Edinburgh Postpartum Depression Scale; FFQ, food frequency questionnaire; m, months; LOT, Life Orientation Test; MED, Mediterranean diet; PSS, Perceived Stress Scale; RCT, randomized controlled trial; y, years.

**Table 3:** Studies evaluating associations between the Mediterranean diet and maternal anthropometric outcomes.

No.	Author and Year	Study Design, Name	Study Size, Country	Sample Characteristic (Special Features, Age)	Dietary Assessment/Intervention, Timing	Mediterranean Diet Assessment	Outcome (Measures, Methods, Timing of Assessment)	Effect of the Mediterranean Diet
1	Stendell-Hollis et al., 2013 [30]	RCT	*n* = 102 USA	74% EBF 27.2–4.9 kg/m^2^ 29.7 ± 4.6 y	MED (28 g walnuts/d, 1–2 tbs EVOO/d) vs. MyPyramid FFQ (153 items), 24-h dietary recall Baseline and 4 m	MED score (9 components)	Anthropometry (BIA) and metabolic outcomes Baseline (2 wk) and after intervention (6 m)	↓ weight, BMI, WC, hip circumference, WHtR, %FM
2	de la Torre et al., 2019 [31]	Intervention study	*n* = 384 (postpartum) Spain	18% GDM 33.2 ± 4.9 y	Educational intervention (12–14 Hbd): >40 mL EVOO and/or nuts	MEDAS (14 components)	Maternal anthropometry and metabolic outcomes 12–14 wk post-partum	↔ weight, BMI, WC
3	Radwan et al., 2021 [32]	Observational study MISC	*n* = 150 United Arab Emirates	98% BF for 2 m 84% BF for 6 m	FFQ (86 items) 3rd trimester	aMED (8 components), LMD (9 components)	Weight changes across pregnancy and postpartum period 2 and 6 m postpartum	↓ PPWR
4	Tabasso et al., 2021 [33]	Cross-sectional study	*n* = 147 Italy	33 ± 6.5–34.5 ± 4.3 y	EPIC-ffq 30 ± 10 d postpartum	IMI (11 components)	Anthropometric and body composition assessment (BOD POD) 30 ± 10 d postpartum	↔ weight, BMI ↓ %FM, FMI
5	Melero et al., 2023 [34]	RCT San Carlos Cohort	*n* = 1400 (postpartum) Spain	29–37 y	Educational intervention (12–14 Hbd): >40 mL EVOO and/or nuts	Adapted MEDAS (12 components)	Anthropometry and metabolic outcomes 3 m and 3 y postpartum	3 m postpartum: ↓ BMI 3 y postpartum: ↓ weight, BMI, WC ↔ FM (kg)
6	Flor-Alemany et al., 2023 [35]	RCT GESTAFIT	*n* = 83 Spain	*n* = 43 exercise intervention *n* = 40 control 33.4 ± 4.2 y	FFQ 16 Hbd	Mediterranean Food Pattern (8 components)	Body composition (DXA) 6th wk postpartum	↑ lean mass ↓ FM, android FM, android to gynoid FM
7	Martín-O’Connor et al., 2024 [36]	RCT	*n* = 453 Spain	prepregnancy overweight	Educational intervention (12–14 Hbd): >40 mL EVOO and/or nuts	MEDAS (14 components)	Anthropometry and metabolic outcomes 3 m and 3 y postpartum	3 m postpartum: ↔ BMI, WC, FM 3 y postpartum: ↑ weight loss ↓ WC ≥ 89.5 cm ↔ BMI, WC, FM
8	Sims et al., 2024 [21]	within-subject intervention study	*n* = 13 USA	EBF Obesity 32.8 ± 3.8 y	Med diet (2 ready-to-eat meals + EVOO) 4 weeks 3-day food records, HEI	MedDiet Score (11 components)	Anthropometry (BOD POD) and metabolic outcomes Baseline, 2 wk and 4 wk	↓ weight, BMI, FMI ↔ FFMI

↔ no association/effect; ↑ positive association/effect; ↓ negative association/effect; aMED, Alternate Mediterranean Index; BIA, bioelectrical impedance analysis; BF, breastfeeding; BMI, body mass index; EBF, exclusive breastfeeding; d, days; DXA, dual-energy X-ray absorptiometry; EPIC-ffq, European Prospective Investigation into Cancer and Nutrition Food Frequency Questionnaire; EVOO, extra-virgin olive oil; FFQ, food frequency questionnaire; FM, fat mass; FFMI, fat-free mass index; FMI, fat mass index; GDM, gestational diabetes mellitus; Hbd, gestational weeks; HEI, Healthy Eating Index; IMI, Italian Mediterranean Index; LMD, Lebanese Mediterranean diet; MEDAS, Mediterranean diet adherence screener; MISC, mother and infant study cohort; PPWR, postpartum weight retention; RCT, randomized controlled trial; WC, waist circumference; wk, weeks; WHtR, waist to hip ratio; y, years.

**Table 4:** Studies evaluating associations between the Mediterranean diet and maternal metabolic outcomes.

No.	Author and Year	Study Design	Study Size, Country, Name	Sample Characteristic (Special Features, Age)	Dietary Assessment/ Intervention, Timing	Mediterranean Diet Assessment	Outcome (Measures, Methods, Timing of Assessment)	Effect of the Mediterranean Diet
1	Stendell-Hollis et al., 2013 [30]	RCT	*n* = 102 USA	74% EBF 27.2–4.9 kg/m^2^ 29.7 ± 4.6 y	MED (28 g walnuts/d, 1–2 tbs EVOO/d) vs. MyPyramid FFQ (153 items), 24 h dietary recall Baseline and 4 m	MED score (9 components)	Plasma FAs (GC), inflammatory biomarkers (ELISA) 4 m	↑ LA, ALA, *ω*-3, *ω*-3/*ω*-6 ratio ↓ *ω*-6/*ω*-3 ratio, TNF-α ↔ IL-6
2	de la Torre et al., 2019 [31]	Intervention study	*n* = 384 (postpartum) Spain	18% GDM 33.2 ± 4.9 y	Educational intervention (12–14 Hbd): >40 mL EVOO and/or nuts	MEDAS (14 components)	Metabolic outcomes 12–14 wk postpartum	↔ FG, insulin, HOMA-IR, HbA1c, sBP, C, HDL-C, LDL-C, TG
3	Melero et al., 2023 [34]	RCT San Carlos Cohort	*n* = 1400 (postpartum) Spain	29–37 y	Educational intervention (12–14 Hbd): >40 mL EVOO and/or nuts	Adapted MEDAS (12 components)	Metabolic outcomes 3 m and 3 y postpartum	3 m postpartum: ↓ C, LDL-C, HDL-C, apo-B, CRP, FG, insulin, HOMA-IR 3 m postpartum: ↓ C, LDL-C, apo-B, CRP, insulin, HOMA-IR, dBP, MetS components ↔ glucose
4	Che et al., 2024 [43]	Cohort Boston Birth	*n* = 1410 USA	28.2 (22.9–33.5) y	21–72 h postpartum	MedDiet Score	Plasma metabolite profile (LC-MS) 24–72 h postpartum	↑ triacylglycerols, phosphatidylcholines plasmalogen, phosphatidylserine, phosphatidylethanolamine, methionine sulfoxide, VA ↓ threonine, tropane, adenosine
5	Martín-O’Connor et al., 2024 [36]	RCT	*n* = 453 Spain	prepregnancy overweightness	Educational intervention (12–14 Hbd): >40 mL EVOO and/or nuts	MEDAS (14 components)	Metabolic outcomes 3 m and 3 y postpartum	3 m postpartum: ↓ LDL-C ↔ MetS components 3 y postpartum: ↔ C, HDL-C, TG, sBP, dBP, MetS components ↓ impaired FG
6	Sims et al., 2024 [21]	within-subject intervention study	*n* = 13 USA	EBF Obesity 32.8 ± 3.8 y	MED diet (2 ready-to-eat meals + EVOO) 4 weeks 3-day food records, HEI	MedDiet Score (11 components)	Metabolic outcomes, inflammatory biomarkers Baseline, 2 wk and 4 wk	↓ C, HDL-C, LDL-C, ↔ leptin, insulin, CRP, IL-6, IL-8, TNF-α
7	Zielinska-Pukos et al., 2024a [22]	Case–control BLOOM	*n* = 40 Poland	*n* = 20 NW *n* = 20 OW/OB 32.4 ± 3.9 y	FFQ (61 food items) 15.05 ± 1.2 w	Polish-aMED (8 components)	Serum leptin (ELISA) 15.5 ± 1.2 w	↑ leptin (in NW)

↔ no association/effect; ↑ positive association/effect; ↓ negative association/effect; ALA, alpha-linolenic acid; apo-B, apolipoprotein B; BLOOM, breastmilk and the link to overweightness/obesity and maternal diet; C, cholesterol; CRP, C-reactive protein; dBP, diastolic blood pressure; EBF, exclusive breastfeeding; EVOO, extra-virgin olive oil; FA, fatty acid; FFQ, food frequency questionnaire; FG, fasting glucose; GC, gas chromatography; GDM, gestational diabetes mellitus; HbA1c, hemoglobin 1Ac (glycated hemoglobin); Hbd, gestational weeks; HDL-C, high-density lipoprotein cholesterol; HEI, Healthy Eating Index; HOMA-IR, homeostatic model assessment for insulin resistance; IL, interleukin; LA, linoleic acid; LC-MS, liquid chromatography–mass spectrometry; LDL-C, low-density lipoprotein cholesterol; MED, Mediterranean diet; MEDAS, Mediterranean diet adherence screener; MetS, metabolic syndrome; NW, normal weight; OW/OB, overweightness/obesity; RCT, randomized controlled trial; sBP, systolic blood pressure; TG, triglyceride; TNF-α, tumor necrosis factor α; wk, weeks; y, years; VA, vitamin A.

**Table 5:** Studies evaluating associations between the Mediterranean diet and lactation and human milk outcomes.

No.	Author and Year	Study Design, Name	Study Size, Country	Sample Characteristic (Special Features, Age)	Dietary Assessment/Intervention, Timing	Mediterranean Diet Assessment	Human Milk Collection Protocol	Outcome (Measures, Methods, Timing of Assessment)	Effect of the Mediterranean Diet
1	Antasouras et al., 2023 [49]	Cross-sectional study	*n* = 5688 Greece	35.1 ± 4.8 y	The third trimester of pregnancy	MedDiet Score (11 components)	N/A	Rates of EBF	↑ EBF for 4 months
**Human milk composition**									
1	Krešić et al., 2013 [50]	Cross-sectional study	*n* = 83 Croatia	31.80 ± 4.60 y Fully BF	Two consecutive 24 h recalls 5th to 25th lactation wk	No details	5 mL of post-feeding human milk expressed manually from one breast between 10 and 12 a.m. 5–25 wk of lactation	FAs profile (GC)	↑ LA, DHA, OA, palmitic acid ↔ AA
2	Codini et al., 2020 [51]	Cross-sectional study	*n* = 30 Italy	31.7 ± 5.1 y Healthy milk donors	No	Evaluated by interview (no details)	Standardized procedure (no details) Samples were submitted to Holder pasteurization	FA profile (GC), antioxidant potential (ORAC)	↑ SFA, PUFA, antioxidant potential
3	Sánchez et al., 2020 [48]	Observational cross-sectional study	*n* = 75 Spain	35.5 ± 4.0 y	No 0.8–59 m postpartum	MEDAS (14 components)	25 mL of prefeed milk was expressed (manually/using a breast pump). No details on the time of collection 0.8–59 m postpartum	Mineral composition (ICP-MS)	↑ Se ↔ Na, K, Ca, Ca/P, Mg, Fe
4	Sánchez-Hernández et al., 2021 [52]	Observational cross-sectional study	*n* = 18 Spain	31.4 (23.0–39.0) y Participants with medium and high adherence	No	MED questionnaire (14 components)	Colostrum (1–5 days, Marmet manual extraction technique). Transitional milk (6–15 days) and mature milk (after 15 days) pre- and post-feed milk was expressed by pump	Phenolic compounds (UPLC-MS/MS), total phenolic compound (Folin assay), antioxidant activity (DPPH, ABTS, FRAP)	↑ total phenolic compound, antioxidant activity
5	Di Maso et al., 2022 [20]	Observational study MEDIDIET	*n* = 282 Italy	33 ± 4 y EBF 6 ± 1 wk postpartum	FFQ (78 items)	MedDiet Score (9 components)	30–50 mL of foremilk expressed using a breast pump after breakfast and before lunch, from 1 to 3 h after a previous BF session 6 ± 1 wk postpartum	FA profile (GC)	↓ SFA, palmitic acid, stearic acid, AA, *ω*-6/*ω*-3, LA/ALA ↑ MUFA, OA, erucic, PUFA *ω*-3, ALA, EPA, DHA, DPA, DHA/AA
6	Karbasi et al., 2023 [23]	Cross-sectional study	*n* = 350 Iran	Mother: 29.5 ± 5.9 y Infants: 1–6 m	FFQ (65 items)	MedDiet Score (8 components)	2 × 20 mL of pre-feeding human milk manually expressed between 7 and 10 a.m. 1–6 m	Protein (Bradford protein assay), TGs and Ca (colorimetric method), total antioxidant activity (FRAP, DPPH, TBARS)	↑ protein, DPPH, FRAP ↓ triglycerides ↔ Ca, thiol
7	Sims et al., 2024 [21]	within-subject intervention study	*n* = 13 USA	EBF Obesity 32.8 ± 3.8 y *n* = 8 male infants	MED diet (2 ready-to-eat meals + EVOO) 4 weeks 3-day food records, HEI	MedDiet Score (11 components)	4 mL of milk from full breast across all feedings across 24 h pooled into one sample Before each study visit	Macronutrients and energy value (MIRIS HM Analyzer), Leptin insulin, CRP, IL-6, IL-8, TNF-α (ELISA) HMOs (HPLC, pre- and post-intervention milk)	↔ macronutrients, energy value, HMOs, insulin, IL-6, CRP ↓ leptin, IL-8, TNF-α
8	Zielinska-Pukos et al., 2024a [22]	Case–control BLOOM	*n* = 40 Poland	*n* = 20 NW *n* = 20 OW/OB 32.4 ± 3.9 y	FFQ (61 food items) 15.05 ± 1.2 w	Polish-Amed (8 components)	An equal volume of pre- and post-feeding milk was expressed during one feeding from four time periods across 24 h 15.05 ± 1.2 w	Leptin in skimmed milk (ELISA)	↓ leptin (in OW/OB)
9	Zielinska-Pukos et al., 2024b [44]	Observational study	*n* = 43 Poland	31.3 ± 3.6 y	FFQ (61 items) First month of lactation	Polish-aMED Scores (8 components)	An equal volume of pre- and post-feeding milk was expressed during one feeding from four time periods across 24 h First month of lactation	Mineral composition (ICP-MS)	↑ Ca, Zn ↔ Fe, P

↔ no association/effect; ↑ positive association/effect; ↓ negative association/effect; AA, arachidonic acid; ALA, alpha-linolenic acid; aMED, alternate Mediterranean Diet Score; BLOOM, breastmilk and the link to overweightness/obesity and maternal diet; BF, breastfeeding; Ca, calcium; CRP, C-reactive protein; d, days; DPPH, 2,2-Diphenyl-1-picrylhydrazyl; EBF, exclusive breastfeeding; ELISA, enzyme-linked immunosorbent assay; EVOO, extra-virgin olive oil; FA, fatty acid; Fe, iron; FFQ, food frequency questionnaire; FRAP, ferric reducing antioxidant power; GC, gas chromatography; HEI, Healthy Eating Index; HMOs, human milk oligosaccharides; HPLC, high-performance liquid chromatography; IL, interleukin; ICP-MS, inductively coupled plasma mass spectrometry; K, potassium; LA, linoleic acid; MED, Mediterranean diet; MEDAS, Mediterranean diet adherence screener; Mg, magnesium; MUFA, monounsaturated fatty acids; Na, sodium; N/A, not applicable; NW, normal weight; OA, oleic acid; OW/OB, overweight/obesity; P, phosphor; PUFA, polyunsaturated fatty acids; SFA, saturated fatty acids; Se, selenium; TBARS, thiobarbituric acid reactive substances; TG, triglyceride; TNF-α, tumor necrosis factor α; UPLC-MS/MS, ultra performance liquid chromatography–tandem mass spectrometry; wk, weeks; y, years; Zn, zinc.

**Table 6:** Studies evaluating associations between the Mediterranean diet and infant outcomes.

No.	Author and Year	Study Design, Name	Study Size, Country	Sample Characteristic (Special Features, Age)	Dietary Assessment/Intervention, Timing	Mediterranean Diet Assessment	Outcome (Measures, Methods, Timing of Assessment)	Effect of the Mediterranean Diet
1	Karbasi et al., 2023 [23]	Cross-sectional study	*n* = 350 Iran	Mother: 29.5 ± 5.9 y Infants: 1–6 m	FFQ (65 items)	MedDiet Score (8 components) 1–6 m	Infant morning urine anti-oxidant status (DPPH, FRAP) 1–6 m of life	↑ urine antioxidants
2	Sims et al., 2024 [21]	within-subject intervention study	*n* = 13 USA	EBF Obesity 32.8 ± 3.8 y *n* = 8 male infants	MED diet (2 ready-to-eat meals + EVOO) 4 weeks 3-day food records, HEI	MedDiet Score (11 components)	Infant anthropometry and body composition (quantitative nuclear magnetic resonance) baseline, 2 wk, and 4 wk of the intervention	↔ weight-for-age, length-for-age, weight-for-length z-scores, FFMI, FMI
3	Grabowski et al., 2024 [54]	Prospective observational study, ABC Baby	N = 167 USA	31.6 ± 4.5 y 49% male infants	FFQ (135 items) 2 wk or 2 m postpartum	MedDiet Score (two versions: 8 and 9 components)	Infant anthropometry (WHO standards), flank skinfold thickness 6 m	↔ weight-for-age, length-for-age ↓ flank skinfold thickness

↔ no association/effect; ↑ positive association/effect; ↓ negative association/effect; d, days; DPPH, 2,2-Diphenyl-1-picrylhydrazyl; EBF, exclusive breastfeeding; EVOO, extra-virgin olive oil; FFQ, food frequency questionnaire; FFMI, fat-free mass index; FMI, fat mass index; FRAP, ferric reducing antioxidant power; HEI, Healthy Eating Index; MED, Mediterranean diet; WHO, World Health Organization; y, years.

## Supplementary Materials

The following supporting information can be downloaded at: https://www.mdpi.com/article/10.3390/nu17111760/s1, Table S1: Mediterranean diet components used in the studies and variables controlled in statistical analyses.

## Abbreviations

The following abbreviations are used in this manuscript:

AA	arachidonic acid
ALA	alpha-linolenic acid
aMED	alternate Mediterranean diet score
AP-Q	adherence to the healthy food pyramid
BIA	bioelectrical impedance analysis
BF	breastfeeding
BLOOM	breastmilk and the link to overweightness/obesity and maternal diet
BMI	body mass index
C	cholesterol
Ca	calcium
CRP	C-reactive protein
d	days
DASH	dietary approaches to stop hypertension
dBP	diastolic blood pressure
DHA	docosahexaenoic acid
DPA	docosapentaenoic acid
DPPH	2,2-Diphenyl-1-picrylhydrazyl
DXA	dual-energy X-ray absorptiometry
EBF	exclusive breastfeeding
ELISA	enzyme-linked immunosorbent assay
EPA	eicosapentaenoic acid
EPDS	Edinburgh Postpartum Depression Scale
EPIC-ffq	European Prospective Investigation into Cancer and Nutrition Food Frequency Questionnaire
EVOO	extra-virgin olive oil
FA	fatty acid
Fe	iron
FFMI	fat-free mass index
FFQ	food frequency questionnaire
FG	fasting glucose
FM	fat mass
FMI	fat mass index
FRAP	ferric reducing antioxidant power
GC	gas chromatography
GDM	gestational diabetes
GWG	gestational weight gain
Hb1Ac	hemoglobin 1Ac (glycated hemoglobin)
Hbd	gestational weeks
HDL-C	high-density lipoprotein cholesterol
HEI	Healthy Eating Index
HM	human milk
HMOs	human milk oligosaccharides
HOMA-IR	homeostatic model assessment for insulin resistance
HPLC	high-performance liquid chromatography
ICP-MS	inductively coupled plasma mass spectrometry
IL	interleukin
IMI	Italian Mediterranean Index
K	potassium
LA	linoleic acid
LC-MS	liquid chromatography–mass spectrometry
LDL-C	low-density lipoprotein
LMD	Lebanese Mediterranean diet
LOT	Life Orientation Test
m	months
MED	Mediterranean diet
MEDAS	Mediterranean Diet adherence screener
Mg	magnesium
MetS	metabolic syndrome
MISC	mother and infant study cohort
MUFA	monounsaturated fatty acids
Na	sodium
NW	normal weight
OA	oleic acid
OW/OB	overweightness/obesity
P	phosphor
PPWR	postpartum weight retention
PSS	Perceived Stress Scale
PUFA	polyunsaturated fatty acids
RCT	randomized controlled trial
sBP	systolic blood pressure
Se	selenium
SFA	saturated fatty acids
TBARS	thiobarbituric acid reactive substances
TG	triglyceride
TNF-α	tumor necrosis factor α
UPLC-MS/MS	ultra-performance liquid chromatography–tandem mass spectrometry
VA	vitamin A
WC	waist circumference
WHO	World Health Organization
WHtR	waist-to-hip ratio
y	years
Zn	zinc
