# Supplementary material for: Mediterranean Diet Outcomes Across the Mother–Milk–Infant Triad: A Narrative Review
Source: Nutrients. 2025 May 23;17(11):1760. doi: 10.3390/nu17111760 (PMC12157837; doi:10.3390/nu17111760)
Supplement: Supplementary file 1 [file nutrients-17-01760-s001.zip › nutrients-3609542-supplementary.pdf]

nutrients

MDPI

Review

Mediterranean Diet Outcomes Across the Mother–Milk–Infant Triad: A Narrative Review

Rabia Baglayici [1,2], Jadwiga Hamulka [1] and Monika A. Zielinska-Pukos [1,*]

[1] Department of Human Nutrition, Institute of Human Nutrition Sciences, Warsaw University of Life Sciences (SGGW-WULS), 02-776 Warsaw, Poland; rabia.baglayici76@gmail.com (R.B.); jadwiga_hamulka@sggw.edu.pl (J.H.)

[2] Department of Nutrition and Dietetics, Institute of Health Sciences, Marmara University, Istanbul 34865, Turkey

* Correspondence: monika_zielinska_pukos@sggw.edu.pl; Tel.: +48-22-593-71-25

Table S1. Mediterranean Diet Components Used in the Studies and Covariates Controlled in Statistical Analyses.

Author	Mediterranean Diet Component	Covariates İncluded in Statistical Analyses
Antasouras et al., 2023 [49]	MedDiet Score based on Panagiotakos et al. [28]: 11 dietary components (cereals, potatoes, fruits, vegetables, dairy products, olive oil, legumes, fish, red meat, and poultry; wine question eliminated)	Maternal age, educational and economic status, nationality, type of residence, smoking habits, parity, pre-pregnancy BMI status, GWG, preterm birth, GDM, gestational hypertension, type of delivery, EBF
Che et al., 2024 [43]	MED index based on Rhee et al. [45]: 10 food components (green vegetables, orange vegetables, fruits, meat, fish, eggs, beans, rice, dairy, and wheat (pasta, bread, and cereal), meat)	Maternal age at delivery, race and ethnicity, education, smoking status during pregnancy, parity, prepregnancy BMI, diabetes status in pregnancy, hypertensive disorders of pregnancy, and gestational age at delivery
Codini et al., 2020 [51]	NA	Tem/preterm delivery
de la Torre et al., 2019 [31]	MEDAS based on Schröder et al. [42]: 14 dietary components (olive oil, vegetables, fruits, legumes, fish, nuts, whole grains, and white meat, along with limited intake of red and processed meat, sugary drinks, and fast food)	NA (secondary comparision of intervention effectiveness between mothers with and without GDM)
Di Maso et al., 2022 [20]	MedDiet Score based on Trichopoulou et al. [37]: 9 dietary components (vegetables, fruit, cereals (including bread and potatoes), legumes, MUFA to SFA ratio (MUFA/SFA) as a proxy of olive oil consumption, fish, dairy products (including milk), meat (including poultry, red and processed meat), and alcohol)	Maternal energy intake per kg of pre-pregnancy weight (kcal/day/kg)
Flor-Alemany et al., 2022 [25]	Mediterranean Food Pattern based on Martínez-González et al. [27]: 8 dietary components (olive oil, fiber, fruits, vegetables, pulses, fish, cereals, meat, and alcohol question eliminated)	Age, prior diagnosis of depression/anxiety, number of abortions, smoking, gestational weight gain, lumbar pain, marital status, educational level. Using stepwise method number of abortions was selected as a significant covariate.
Flor-Alemany et al., 2023 [35]	MedDiet Score based on Panagiotakos et al. [28]: 11 dietary components (wholegrain cereals, potatoes, fruits, vegetables, pulses, fish, olive oil, red wine, red meat and subproducts, poultry, and whole dairy products. Alcohol question eliminated)	Age, pre-pregnancy BMI
Gila-Díaz et al., 2021 [24]	The AP-Q based on Gila-Díaz et al. [29]: 11 food components (grains; seeds and legumes; fruits;	Unadjusted correlations

 https://doi.org/10.3390/xxxxx

	vegetables; oil type; dairy products; animal proteins; and snacks) In total 27 multiple-choice questions grouped into 10 different categories, including physical activity; healthy habits (lifestyle, emotional balance, sleep hygiene, and culinary techniques) and culinary techniques; hydration (water intake, soft drinks, wine and beers, and distilled beverages)	
Grabowski et al., 2024 [54]	MedDiet Score based on Fung et al. [46]: 8 dietary components (vegetables (excluding potatoes), fruits, whole grains, nuts and seeds, legumes, fish, monounsaturated fat (MUFA)-to-saturated fat (SFA) ratio (MUFA:SFA), and red and processed meat) Second version - included added sugar as a component	Maternal BMI at baseline, infant birth weight, breastfeeding intensity at 2 months. Sensitivity analyses included maternal education level and maternal age
Karbasi et al., 2023 [23]	MedDiet Score based on Trichopoulou et al. [37]: 8 dietary components (fruits, vegetables, nuts, grain, legumes, fish, and seafood, the ratio of monounsaturated to saturated fatty acids (MUFA/SFA), meat and dairy products, alcohol question eliminated)	Age, BMI, and energy intake, sBP, dBP; infant age, sex, weight, and head circumference (depending on analysis)
Krešić et al., 2013 [50]	NA	NA
Martín-O'Connor et al., 2024 [36]	MEDAS based on Schröder et al. [42]: 14 dietary components (olive oil, vegetables, fruits, legumes, fish, nuts, whole grains, and white meat, along with limited intake of red and processed meat, sugary drinks, and fast food)	Not specified
Melero et al., 2023 [34]	MEDAS based on Schröder et al. [42]: 12 dietary components (olive oil, vegetables, fruits, legumes, fish, nuts, whole grains, and white meat, along with limited intake of red and processed meat, sugary drinks, and fast food; the alcohol and juices question eliminated)	Maternal age, pre-pregnancy BMI
Papadopoulou et al., 2023 [26]	MedDiet Score based on Panagiotakos et al. [28]: 11 dietary components (cereals, potatoes, fruits, vegetables, dairy products, and olive oil, legumes, fish, red meat, and poultry	Pre-pregnancy and postpartum BMI, GWG, preterm birth, type of delivery, EBF, maternal age, education, socioeconomic status, nationality, marital status, employement status, smoking (depending on analysis)
Radwan et al., 2021 [32]	aMED based on Trichopoulou et. al [37] and de Koning et al. [38]: (8 dietary components (whole grains, MUFA:SFA ratio, fruits, vegetables, legumes, nuts, fish and seafood, red and processed meats) LMD based on Naja et al. [40]: 9 dietary components (whole grains/bulgur, olive oil, fruits, dried fruits, vegetables, starchy vegetables, legumes, eggs, as well as milk and dairy products)	Maternal age, parity, energy intake
Sánchez et al., 2020 [48]	MEDAS based on Schröder et al. [42] and Martínez-González et al. [53]: 14 dietary components (olive oil, vegetables, fruits, legumes, fish, nuts, whole grains, and white meat, along with limited intake of red and processed meat, sugary drinks, and fast food)	Lactation duration, BMI, maternal age, newborn sex.
Sánchez-Hernández et al., 2021 [52]	MEDAS based on Schröder et al. [42] and Martínez-González et al. [53]: 14 dietary components (olive oil, vegetables, fruits, legumes, fish, nuts, whole grains, and white meat, along with limited intake of red and processed meat, sugary drinks, and fast food)	NA (all participants exhibit medium-to-high adherence to a MED)
Sims et al., 2024 [21]	MedDiet Score based on Goulet et al. [39]: 11 dietary components (grains, fruits, vegetables, legumes, nuts	NA (comparison of outcomes at baseline and 4 wk of intervention)

	and seeds, olive oil, dairy products, fish, poultry, eggs, sweets and red meat/processed meat)	
Stendell-Hollis et al., 2013 [30]	MED score based on Trichopoulou et. al [37]: 9 dietary components (vegetables, legumes, fruits and nuts, whole grain cereals, fish, meat/poultry, dairy, MUFA/ SAT ratio, and ethanol)	NA (comparison of outcomes at baseline and 4 wk of intervention
Tabasso et al., 2021 [33]	IMI based on Agnoli et al. [41]: 11 dietary components (vegetables, fruits, legumes, olive oil, fish, red and processed meat, potatoes, butter, soft drinks and alcohol)	NA (comparision groups: IMI <5 points vs.IMI ≥ 5 points)
Zielinska-Pukos et al., 2024 [22]	Polish-aMED based on Fung et al. [46] and Krusinska et al. [47]: 8 dietary components (vegetables, fruit, wholegrains, fish, legumes, nuts and seeds, ratio of vegetable oils to animal fat, red and processed meat)	Maternal age, FM%
Zielinska-Pukos et al., 2024 [44]	Polish-aMED based on Fung et al. [46] and Krusinska et al. [47]: 8 dietary components (vegetables, fruit, wholegrains, fish, legumes, nuts and seeds, ratio of vegetable oils to animal fat, red and processed meat)	Lactation duration, maternal age

Specific references were listed in the main document. aMED, alternate Mediterranean Diet; AP-Q, Adherence to the Healthy Food Pyramid; BMI, body mass index; dBP: diastolic blood pressure; EBF, exclusive breastfeeding; FM%, fat mass percentage; GDM, gestational diabetes mellitus; GWG, gestational weight gain; IMI, Italian Mediterranean Index; LMD, Lebanese Mediterranean Diet; MED, Mediterranean Diet; MEDAS, Mediterranean Diet Adherence Screener; MUFA, monounsaturated fatty acids; NA, not Available; sBP, systolic blood pressure; SFA, saturated fatty acids; wk, weeks.
